# Supplementary material for: Energy Density and Level of Processing of Packaged Food and Beverages Intended for Consumption by Australian Children
Source: Nutrients. 2025 Jul 11;17(14):2293. doi: 10.3390/nu17142293 (PMC12299509; doi:10.3390/nu17142293)
Supplement: Supplementary file 1 [file nutrients-17-02293-s001.zip › Table S1.pdf]

Table S1: Common ingredients identified as markers of ultra-processed (MUP) foods used to classify products as G4 (43).

MARKERS OF ULTRA-PROCESSED FOODS (UPFs)(G4) *		
**Ingredient**		**Purpose/Reason**
Maltodextrin		Bulking agent/thickener/sweetener/industrially derived substance/cosmetic additive
Dextrin		Emulsifier/thickener
Wheat gluten		Bread improver
Collagen		Industrial food substance
Humectant		Cosmetic additive (texture and moisture control)
Glycerine/glycerin		Humectant/thickener
Glycerol		Humectant/thickener
Gelatin/gelatine		Gelling agent/emulsifier/thickener
Industrially derived sweeteners:		
Glucose	Dextrose	Industrially derived sweetener
Glucose syrup	Golden syrup	
Wheat glucose syrup	Invert sugar	
Dried glucose syrup	Agave syrup	
Fructose	Rice malt syrup	
High fructose corn syrup	Corn syrup	
Non-nutritive sweetener:		
Natural sweetener	Acesulphame potassium	Non-nutritive sweetener
Sweetener	Aspartame	
Malitol	Sucralose	
Stevia	Thaumatin	
Stevia leaf extract	Xylitol	
Steviol glycosides	Neotame	
Steviol	Mannitol	
Erthritol		
Gums:		
Vegetable gum	Guar gum	Gelling /thickening agent
Xanthan gum	Pectin	
Locust bean gum	Agar	
Natural gum		
Extracts:		
Mustard extract	Rosemary extract	Flavour/cosmetic additive
Spice extract	Aloe vera extract	
Herb extract	Tamarind extract	
Yeast extract		
Protein isolates:		
Whey	Hydrolysed vegetable protein (HVP)	Protein isolate
Whey powder	Hydrolysed soy protein	
Protein	Hydrolysed plant protein	
Milk protein	Textured vegetable protein	
Soy protein	Pork protein	
Hydrolysed maize protein		
Emulsifiers:		
Diphosphates	Polyglycerol polyricinoleate	Cosmetic additive/industrial derived food substance/emulsifier
Distilled monoglyceride	Soy lecithin	
Mono and diglycerides	Sunflower lecithin	
Mono and diglycerides of fatty acids		
Thickeners:		
Carrageenan	Vegetable derived mono-glyceride	Cosmetic additive/thickener
Concentrates:		
Concentrated juice	Juice concentrate	Sweetener/industrial food substance
Modified starches:		
Modified corn starch	Modified tapioca starch	Thickener/industrial derived food substance

Colours:<br>Natural colour<br>Natural colour (carmine)<br>Natural colour (turmeric)<br>Natural colour (paprika)<br>Caramel (colour)<br>Caramel I<br>Tartarzine<br>Ferrous gluconate	 Carmine<br>Annatto extracts<br>Beta-carotene<br>Carotene<br>Paprika oleosin<br>Paprika extract<br>Turmeric oleoresin<br>Chlorophyll	 Colour/cosmetic additive
Flavours:<br>Natural flavour<br>Vanilla flavour<br>Vanilla extract<br>Barbeque flavour<br>Vegetable extract	 Smoke flavour<br>Natural flavour<br>Natural chicken flavour<br>Natural banana flavour	 Flavour/cosmetic additive
Flavour enhancers:<br>MSG<br>Monosodium glutamate<br><br>Monosodium L-glutamate	 Magnesium glutamate<br>Monopotassium L-glutamate<br>Maltol	 Flavour enhancer
Isolated fibres:<br>Dietary fibre<br>Fibre<br>Wheat fibre<br>Oat fibre<br>Citrus fibre	 Inulin/GOS/FOS<br>Polydextrose<br>Potato fibre<br>Barley beta glucans<br>Chicory	 <br>Isolated fibre/industrial food substance
Anti-caking agents:<br>Cellulose		 Anti-caking agent
Hydrogenated oils:<br>Hydrogenated palm oil	 Shortening	 Hydrogenated oil
Margarine		UPF produced through multiple industrial processes
Bran straws		Industrial food substance
Alcoholic liqueurs		Distilled alcohol
Alcoholic spirits		Distilled alcohol
Sherry		Fortified wine
Brandy		Fortified wine
Aromas		Cosmetic additive
Aroms of truffle		Cosmetic additive
Fish sauce		Usually contains MUPs
Oyster sauce		Usually contains MUPs
Tamari sauce		Usually contains MUPs
Seasonings		Usually contains MUPs
Stocks		Usually contains MUPs

**This is not an extensive list of MUPs but includes most commonly used MUPs. UPF; ultra-processed food, MUP; markers of ultra-processed foods, G4; ultra-processed food.*
