# Supplementary material for: Energy Density and Level of Processing of Packaged Food and Beverages Intended for Consumption by Australian Children
Source: Nutrients. 2025 Jul 11;17(14):2293. doi: 10.3390/nu17142293 (PMC12299509; doi:10.3390/nu17142293)
Supplement: Supplementary file 1 [file nutrients-17-02293-s001.zip › Table S2. Descriptive data of ED for Total Sample.pdf]

Table S2: Descriptive data for each 'Food' and 'Drink' subcategory by group ('Total Sample') reported by Mintel Global New Products Database as being released in Australia (2013-2023).

	'Total Sample'							
	n				ED (kJ/g or ml)			
FOOD	Total (n)	Low ED	Med ED	High ED	Median	IQR	Min	Max
Baby Food*	520	329	30	161	3.23	12.38	1.28	23.80
Bakery	167	0	23	144	18.36	4.27	8.07	22.46
Breakfast Cereals	83	1	1	81	16.00	0.66	4.11	19.70
Chocolate Confectionary	97	0	0	97	22.41	1.52	16.27	24.87
Dairy*	28	12	6	10	9.15	10.41	3.40	17.90
Desserts & Ice Cream	91	40	50	1	4.64	3.31	0.06	13.00
Fruit & Vegetables	7	7	0	0	1.88	1.98	1.38	4.06
Meals & Meal Centres	17	11	6	0	3.10	1.88	2.32	9.56
Processed Fish, Meat & Pork Products	19	0	19	0	7.46	1.61	4.24	9.83
Sauces & Seasonings	4	4	0	0	3.14	0.59	3.00	4.05
Savoury Spreads	5	0	3	2	11.15	5.57	6.40	17.05
Side Dishes	2	1	1	0	5.58	4.75	3.20	7.95
Snacks	247	22	12	213	16.26	4.47	2.33	25.56
Sugar & Gum Confectionary	195	2	9	184	14.50	1.46	1.43	20.07
Sweet Spreads	3	0	0	3	14.20	8.00	14.13	22.13
**'Food' Total**	**1485**	**429**	**160**	**896**	**14.44**	**13.81**	**0.06**	**25.56**
DRINK	Total (n)	Low ED	Med ED	High ED	Median	IQR	Min	Max
Baby Food*	100	100	0	0	2.82	0.27	0	28.00
Carbonated Soft Drinks	7	7	0	0	1.94	1.30	0.75	2.24
Dairy*	87	0	1	86	2.94	1.11	1.84	4.21
Hot Beverages	5	1	0	4	22.60	0	2.00	22.60
Juice Drinks	46	46	0	0	1.23	0.62	0.59	2.86
Nutritional Drinks & Other Beverages	34	24	5	5	15.15	2.41	0.12	19.13
Water	6	6	0	0	0.01	0.32	0.00	0.33
**'Drink' Total**	**285**	**184**	**6**	**95**	**2.74**	**1.07**	**0**	**22.60**
**Total**	**1770**	**613**	**166**	**991**	**13.30**	**13.80**	**0**	**25.56**

** Subcategory includes some liquid products that Mintel Global New Products Database considered as 'Food' that was reclassified as 'Drink' due to the way they are consumed. e.g. 'Food' subcategory Baby Food products such as Growing Up Milks were reclassified under 'Drink' Baby Food and Drinkable Yoghurt & Liquid Cultures, Flavoured Milk, Plant Based Drink and White Milk under 'Food' Dairy subcategory were reclassified under 'Drink' Dairy subcategory. n; number of items, ED; energy density, IQR; interquartile range. ED classified as low (≦4.184kJ/g), medium (>4.184kJ/g and <12.552 kJ/g), or high (≧12.552 kJ/g).*
